# Distinct Expression Patterns of Interleukin-22 Receptor 1 on Blood Hematopoietic Cells in SARS-CoV-2 Infection

**DOI:** 10.3389/fimmu.2022.769839

**Published:** 2022-03-29

**Authors:** Nurhan Albayrak, Carmen Orte Cano, Sina Karimi, David Dogahe, Anne Van Praet, Audrey Godefroid, Véronique Del Marmol, David Grimaldi, Benjamin Bondue, Jean-Paul Van Vooren, Françoise Mascart, Véronique Corbière

**Affiliations:** ^1^ Laboratory of Vaccinology and Mucosal Immunity, Université Libre de Bruxelles, Brussels, Belgium; ^2^ Department of Dermatology, Hopital Erasme, Université Libre de Bruxelles, Brussels, Belgium; ^3^ Department of Internal Medicine, Hopital Erasme, Université Libre de Bruxelles, Brussels, Belgium; ^4^ Department of Intensive Care Unit, Hopital Erasme, Université Libre de Bruxelles, Brussels, Belgium; ^5^ Department of Pneumology, Hopital Erasme, Université Libre de Bruxelles, Brussels, Belgium; ^6^ Immunodeficiency Unit, Hopital Erasme, Université Libre de Bruxelles, Brussels, Belgium

**Keywords:** SARS-CoV-2 infection, COVID-19, interleukin-22, interleukin-22 receptor, monocytes, dendritic cells, NK cells, T lymphocytes

## Abstract

The new pandemic virus SARS-CoV-2 is characterized by uncontrolled hyper-inflammation in severe cases. As the IL-22/IL-22R1 axis was reported to be involved in inflammation during viral infections, we characterized the expression of IL-22 receptor1, IL-22 and IL-22 binding protein in COVID-19 patients. Blood samples were collected from 19 non-severe and 14 severe patients on the day they presented (D0), at D14, and six months later, and from 6 non-infected controls. The IL-22R1 expression was characterized by flow cytometry. Results were related to HLA-DR expression of myeloid cells, to plasma concentrations of different cytokines and chemokines and NK cells and T lymphocytes functions characterized by their IFN-γ, IL-22, IL-17A, granzyme B and perforin content. The numbers of IL-22R1^+^ classical, intermediate, and non-classical monocytes and the proportions of IL-22R1^+^ plasmacytoid DC (pDC), myeloid DC1 and DC2 (mDC1, mDC2) were higher in patients than controls at D0. The proportions of IL-22R1^+^ classical and intermediate monocytes, and pDC and mDC2 remained high for six months. High proportions of IL-22R1^+^ non-classical monocytes and mDC2 displayed HLA-DR^high^ expression and were thus activated. Multivariate analysis for all IL-22R1^+^ myeloid cells discriminated the severity of the disease (AUC=0.9023). However, correlation analysis between IL-22R1^+^ cell subsets and plasma chemokine concentrations suggested pro-inflammatory effects of some subsets and protective effects of others. The numbers of IL-22R1^+^ classical monocytes and pDC were positively correlated with pro-inflammatory chemokines MCP-1 and IP-10 in severe infections, whereas IL-22R1^+^ intermediate monocytes were negatively correlated with IL-6, IFN-α and CRP in non-severe infections. Moreover, in the absence of *in vitro* stimulation, NK and CD4^+^ T cells produced IFN-γ and IL-22, and CD4^+^ and CD8^+^ T cells produced IL-17A. CD4^+^ T lymphocytes also expressed IL-22R1, the density of its expression defining two different functional subsets. In conclusion, we provide the first evidence that SARS-CoV-2 infection is characterized by an abnormal expression of IL22R1 on blood myeloid cells and CD4^+^ T lymphocytes. Our results suggest that the involvement of the IL-22R1/IL-22 axis could be protective at the beginning of SARS-CoV-2 infection but could shift to a detrimental response over time.

## Introduction

The SARS-CoV-2 is a new coronavirus emerging from China at the end of 2019 and causing the recent COVID-19 infection declared as a pandemic by the World Health Organization (WHO) in March 2020 (1). Clinical spectrums of the COVID-19 range from asymptomatic to mild or severe clinical forms and even death. The reasons for this diversity are still not fully understood (2, 3). Whereas direct cytopathic effects are thought to play a significant role in COVID-19 pathology, dysregulated host immune responses like low HLA-DR expression on myeloid cells or impaired cell functions have also been suggested partly responsible for the clinical presentation of severe forms of the disease (4–8). A reduction in the numbers of circulating NK cells, dendritic cells (DC) and T lymphocytes, and/or their impaired functions may directly be responsible for the progression to the severe disease of COVID-19 (5–9). Recent studies also suggest that over-reactive immune responses inducing the uncontrolled hyper-inflammation state may lead to the development of inadequate adaptive immune response, and eventually to lung injury and acute respiratory distress syndrome (ARDS), as well as multisystemic failure (4, 5, 7, 10–12). Nevertheless, the precise mechanisms of these immune dysregulations remain elusive (7, 10).

The role of the interleukin-22 (IL-22)/interleukin-22 receptor (IL-22R) axis is well described in lung tissues, where it is essential for host protective immunity to both bacterial and viral infections (13–15). The heterodimeric receptor IL-22R is composed of IL-22R1 and IL-10β chains, and the former is restricted to non-hematopoietic cells of the lung, skin, pancreas, intestine, liver and kidney in normal physiological conditions (15). However, recent studies reported abnormal expression of the IL-22R on circulating myeloid cells from patients with autoimmune diseases (16), on T cells in hematological malignancies (17, 18), and on *Mycobacterium tuberculosis* infected macrophages (19, 20). The presence of the IL-22R1 determines the target sites of IL-22, which is produced by a wide range of innate and adaptive immune cells such as NK and T cells. Binding to its membrane-bound receptor IL-22R in different tissues leads to epithelial cell proliferation, restoration of epithelial tight junctions, inhibition of apoptosis, secretion of antimicrobial peptides and tissue repair (14, 15, 20). More specifically during viral infections, activation of IL-22R on lung epithelial cells after influenza infection, has been associated with tissue repair and restoration of epithelial integrity (21). IL-22 has also been shown to inhibit porcine coronavirus infection in an intestinal epithelial cell line by upregulating antimicrobial peptide production (22). On the contrary, dysregulated IL-22 responses may lead to pathological inflammation and substantially damage barrier surfaces, mainly in chronic settings (23, 24). IL-22R signaling during viral infection induces several chemokine secretions, which play a role in neutrophil recruitment to the infection site. This might be beneficial or lead to hyper-inflammatory conditions, depending on the virus type involved and the tissue where signaling occurs (14). Due to the dual-natured role of IL-22, the same functions could be beneficial or detrimental depending on the unique inflammatory milieu, likewise, the presence of neutrophils or the concomitant expression of IL-17 (15, 24–27). The possible harmful interaction between IL-22 and IL-22R is tightly regulated by the naturally occurring antagonist IL-22 binding protein (IL-22BP). IL-22BP is constitutively produced by epithelial cells of the lung and some hematopoietic cells and binds to IL-22 with greater affinity than the membrane receptor. Therefore it prevents the biological activation of IL-22 signaling (15, 24, 28, 29).

Thus, we hypothesized that the expression of IL-22R1 on immune cells might be affected by COVID-19 and could be related to the degree of the disease severity. During the first wave of the pandemic, we characterized the IL-22/IL-22R axis at different time points in peripheral blood cells from COVID-19 patients presenting non-severe or severe forms and receiving no other treatment than a supportive one. So, we studied the expression of IL-22R1 on blood hematopoietic cells, HLA-DR expression on IL-22R1 expressing cells, and their association with plasma pro-inflammatory mediators. In parallel, IL-22-producing NK, CD4^+^ and CD8^+^ T cells and their functional characteristics were analyzed.

## Materials and Methods

### Study Groups

COVID-19 suspected patients were recruited prospectively at the University Hospital Erasme, Brussels, Belgium, from April to August 2020 during the first wave of the pandemic. Only patients with SARS-CoV-2 infection confirmed by diagnostic reverse-transcription polymerase chain reaction (RT-PCR) were considered for the analysis. According to WHO guidelines, patients with COVID-19 were classified into either the severe or non-severe groups, including mild and moderate disease, based on their oxygen saturation rates and supplementary oxygenation needs (30). None of these patients received corticosteroid treatment. The study was approved by the ethical committee “Comité d’Ethique hospitalo-facultaire ERASME-ULB” (021/406), and all persons gave written informed consent to participate in the study. Samples from six healthy subjects confirmed as SARS-CoV-2 RT-PCR negative and serology negative were kindly provided by Arnaud Marchant’s team (Institute for Medical Immunology, Université Libre de Bruxelles) and used as a control group for our study and analyzed on whole-blood cells.

### Blood Sampling

Blood samples from COVID-19 patients were collected immediately on the first day the patients presented at the hospital before any treatment (day 0), on day 14, followed by a six-month sample for patients who agreed to come back for an additional sampling (**Table 1**). Patients with more than 14 days delay of symptom onset were not included in the study to prevent the immunological variations. Two patients’ blood samples were taken on day 5, as they were considered as “non-severe disease” at hospital admission but developed severe disease days after. Whole blood was collected by venipuncture using BD Vacutainer Sodium Heparin Tubes (BD Biosciences, Erembodegem, Belgium) and sent to the laboratory within 2 hours. Then, 1 mL whole blood was mixed with a 1 mL whole blood stabilizer (Cytodelics AB, Sweden) and cryopreserved according to the manufacturer’s recommendations until further use for phenotypic characterization of innate immune cells by flow cytometry. In parallel, peripheral blood mononuclear cells (PBMC) were isolated from peripheral blood by density gradient centrifugation over Ficoll-Hypaque using BD Vacutainer^®^ CPT™ for Mononuclear Cell Preparation (BD Bioscience), and they were frozen at −86°C for further use in functional assays. Plasma samples were collected from EDTA tubes after centrifugation for 10 min at 1500 g and were immediately stored in several aliquots at −20°C until their use for ELISA.

**Table 1:** Demographic and clinical characteristics of COVID-19 patients and healthy controls included in the study.

	Healthy	Non-severe	Severe	*p-*value
**Sample numbers**				
Day 0	6	19	14	–
Day 14	–	11	9	–
Month 6	–	8	4	–
**Demographic data**				
Age, median years (min-max)	43 (27-48)	47 (24-63)	45 (36-82)	*^#^
Gender, female (%)	66.7%	44.5%	35.7%	ns
**Symptoms**				
Symptoms onset day (min-max)	–	6.5 (1-14)	7 (2-14)	ns
Fever	–	61.1%	50%	ns
Dyspnea	–	55.6%	83.3%	ns
Cough	–	72.2%	66.7%	ns
**Hospital admission and blood tests on admission** ^##^ **(median; min-max) [sample numbers]**				
Hospitalization time (days)	–	1 (1-14) ^[n=19]^	11.5 (6-50) ^[n=14]^	****
Neutrophiles (mm^3^)	–	3260 (1070-14410) ^[n=14]^	5420 (1970-17160) ^[n=13]^	*
Lymphocytes (mm^3^)	–	1405 (720-2730) ^[n=14]^	950 (420-2520) ^[n=13]^	ns
Neutrophiles/lymphocytes ratio	–	2.02 (0.63-8.48) ^[n=14]^	4.27 (1.44-16.34) ^[n=13]^	*
Creatinin (mg/dL)	–	0.875 (0.57-3.79) ^[n=16]^	0.77 (0.39-12.41) ^[n=13]^	ns
CRP (mg/dL)	–	16.5 (0.05-180) ^[n=18]^	103 (5.1-210) ^[n=14]^	**
**Severity (n,%) ^[sample numbers]^**				
ARDS	–	–	6 (42.9%) ^[n=14]^	–
Oxygen supplementation	–	5 (27.8%) ^[n=19]^	14 (100%) ^[n=14]^	–
Invasive ventilation	–	–	6 (42.9%) ^[n=14]^	–
Death	–	–	2 (14.3%) ^[n=14]^	–
Long-term COVID-19^###^	–	2 (25%) ^[n=8]^	3 (75%) ^[n=4]^	ns
				

ns, not significant; *p<0.05, **p<0.01, and ****p<0.0001.

^#^p values for non-severe and severe COVID-19 compared to healthy controls.

^##^Blood samples from two patients with severe disease were collected on day 5 of their admission in place of day 0, as they initially presented a non-severe disease, and they developed a severe disease at day 5.

^###^Information restricted to patients with a six months follow-up.

### Phenotypic Characterization of Whole Blood Innate Immune Cells

Before flow cytometry staining, cells cryogenically preserved in Cytodelics Stabilizer were thawed using a 37˚C water bath. Then cells were fixed and washed according to the kit procedures after red blood cells lysis (whole blood processing kit; Cytodelics AB, Sweden). All-time points from the same patients were thawed and acquired on the same day to avoid variations due to flow cytometer fluctuations.

Cells contained in a minimum of 500 µl original whole blood were thawed and labelled for 30 minutes at room temperature with the cocktail of monoclonal antibodies (mAbs) consisting of anti-CD45 EF450, anti-CD3 EF506, anti-CD14 FITC, anti-CD16 SB780, anti-CD11c SB645, anti-CD123 SB600, anti-CD141 PE-Cy7, anti-CD56 APC eFluor780, anti-HLA-DR SB-702 and anti-IL-22R PE in the presence of Super Bright Complete Staining Buffer (**Supplementary Table 1A**).

### Functional Characterization of PBMCs

To determine the functional characteristics of NK and T cells, thawed PBMC were used for intracellular flow cytometry staining from three healthy controls, five non-severe and four severe COVID-19 patients. The PBMC from controls were taken from our laboratory’s biobank (BB190012), collected before the pandemic. The PBMC from COVID-19 patients were selected with the highest IL-22R1 expressing profiles. Thawed PBMC were rested during two hours in culture medium, a supplemented RPMI described previously (31), in the presence of 10 U/mL DNAse I (Sigma-Aldrich, Bornem, Belgium). Then, 1x10^6^ cells in 500 µL RPMI were incubated for 24 hours at 37°C and 5% CO2 in a humidified atmosphere in the presence or absence of an NK-activating-interleukin-cocktail composed of IL-12 (10 ng, Biolegend), IL-15 (10 ng, RD Systems) and IL-18 (50 ng, RD Systems) (32). Brefeldin A (10 µg/mL, Sigma-Aldrich) and monensin (1/1000; BD Biosciences) were added during the last 4 hours of incubation.

Following the 24 hours incubation period, cells were characterized for their IL-22, IL-17A, IFN-γ, granzyme B and perforin contents by a two-step intracellular flow cytometry staining. PBMC were first labelled for 30 minutes at +4°C with anti-CD4 APC-Cy7, anti-CD8 BV605, anti-CD14 AF700, anti-CD16 SB780, anti-CD56 APC and anti-IL-22R PE antibodies in the presence of Super Bright Complete Staining Buffer (eBioscience). The cells were further fixed and permeabilized using the BD Cytofix/Cytoperm fixation/permeabilization kit following the manufacturer’s instructions. Finally, the intracellular staining step was performed with anti-CD3 EF506, anti-granzyme B BV421, anti-perforin PerCP Cy5.5 Biolegend), anti-IFN-γ BV711, anti-IL-22 PE-Cy7 and anti-IL-17A FITC antibodies in the presence of the Super Bright Complete Staining Buffer (**Supplementary Table 1B**).

### Flow Cytometry and Data Analysis

Both whole blood cells and PBMCs were acquired on a BD-LSR Fortessa flow cytometer. At least 50,000 cells (events) gated on the lymphocytes or monocytes region were acquired for each sample. Compensations were performed using Comp Beads (BD Bioscience) tubes individually stained with each fluorophore for both panels, and compensation matrices were calculated with FACSdiva. The acquisition data for different monocyte subpopulations, CD14^high^ CD16^neg^ classical monocytes, CD14^high^ CD16^pos^ intermediate monocytes, CD14^low^ CD16^pos^ non-classical monocytes (33); for different dendritic cell (DC) subsets, CD123^pos^ plasmacytoid DC (pDC), CD11c^pos^ CD141^pos^ myeloid DC1 (mDC1) and CD11c^pos^ CD141^neg^ myeloid DC2 (mDC2) (34, 35); for two main natural killer (NK) cell subsets, CD56^bright^ and CD56^dim^ NK cells (5) and the IL-22R1 and HLA-DR expressions were analyzed using FlowJo 9.5.3 software (Tree Star, Ashland, OR USA) (36). The polyfunctional capacity of the cells producing different combinations of the cytokines provided from FlowJo was investigated by Boolean analysis. After doublet exclusions, cell viability was determined *via* Forward and Side Scatter appearance by plotting forward scatter height and forward scatter area. Minimum 100 events for cells and 30 events for the expressions were accepted for the analysis. Absolute cell counts were estimated from whole blood by acquisitions of 50 µl of CountBright™ Absolute Counting Beads (Invitrogen) in each tube. Results were expressed as absolute cell numbers in 1 mL whole blood for the distribution of the cells and cell subsets and as percentages, absolute numbers and Median Fluorescence Intensity (MFI) for IL-22R1. The percentages of HLA-DR^high^ expression was measured for IL-22R1^+^ or IL-22R1^neg^ cells. Results of cytokine content were indicated as percentages of cytokine-containing cells and those of granzyme B and perforin were reported as the MFI of the subset-containing cells. The gating strategy for the phenotypic characterization of whole blood and functional characterization of PBMC is shown in **Supplementary Figures 1**, **2**, respectively.

### ELISA for Measurement of Blood IL-22 and IL-22BP Concentrations

Plasma IL-22 and IL-22BP levels from six healthy controls and 32 patients (18 non-severe, 14 severe) were measured by classical sandwich enzyme-linked immunosorbent assay (ELISA). The Human IL-22 Quantikine ELISA kit (R&D Systems) and the Human IL-22BP DuoSet ELISA kit (R&D Systems) were used according to the manufacturer’s instructions.

### Multiplex ELISA for Measurement of Blood Pro-Inflammatory Cytokine and Chemokine Concentrations

The concentrations of 16 pro-inflammatory cytokines and chemokines were measured in plasma collected at D0 from 6 controls, 10 non-severe and 11 severe COVID-19 patients by multiparameter-based immunoassays (Milliplex Human Cytokine/chemokine/Growth factor panel A magnetic bead panel kit-Merck, Belgium) according to the manufacturer’s instructions. The samples were selected chronologically from patients with a clear status. The panel included granulocyte macrophage colony-stimulation factor (GM-CSF), interferon-alpha (IFN-α), interferon-gamma (IFN-γ), interleukin (IL)-1β, IL-6, IL-7, IL-8, IL-10, IL-17A, IL-22, interferon gamma-induced protein 10 (IP-10), monocyte chemo-attractant protein-1 (MCP-1), monokine induced by gamma interferon (MIG), macrophage inflammatory protein (MIP)-1α, MIP-1β, and tumor necrosis factor alpha (TNF-α). Results were analyzed with a Bio-Plex 200^®^ Multiplex reader, Bio-Plex ManagerTM Manager 4.1 Software (BIO-RAD laboratories, Nazareth Eke, Belgium).

### Statistical Analysis

GraphPad Prism 9 (GraphPad Software, La Jolla, CA, USA) was used for statistical analysis. Statistical differences between groups were calculated using the non-parametric Kruskal Wallis test with Dunn’s test as *post hoc* applied for multiple group comparisons, whereas a 2-tailed Mann-Whitney U test was used to compare single comparisons, independent groups. Significant differences of paired analyses were calculated using 2-tailed Wilcoxon’s matched pairs or Friedman test for paired analysis. The Chi-square test was performed for the gender distribution and the symptoms of patients for the subject groups. Non-parametric Spearman’s rank-order correlation test was utilized to quantify the expression associations. The predictive value for the disease severity of absolute cell numbers of IL-22R1 expressing myeloid cells for each subset as univariate and as multivariate was evaluated after depicting the receiver operating characteristic (ROC) curve of statistically significant variables and calculating the area under the curve (AUC). Results are shown as median, p-values < 0.05 were regarded as significant, and the statistical significances are indicated as *p<0.05, **p<0.01, ***p<0.001 and ****p<0.0001 in the Figures.

## Results

### Demographic and Clinical Characteristics of COVID-19 Patients

Thirty-three PCR-positive COVID-19 patients, comprising 19 with non-severe forms and 14 with severe forms with lung involvement, as well as six healthy non-infected volunteers, were included in the study. COVID-19 patients were recruited during the first wave of the pandemic in Belgium. For COVID-19 patients, blood samples were taken on the day of admission (day 0), on day 14 and six months later from patients who agreed to participate. The primary demographic data and clinical characteristics of the subjects revealed no significant differences between the groups concerning gender, symptoms onset day and nature of symptoms. At the same time, the average age distribution was higher in patients than in healthy individuals. The duration of hospitalization, CRP levels and neutrophil counts were significantly higher in patients with severe compared to non-severe diseases. As some patients with severe disease were lymphopenic, neutrophil/lymphocyte ratios were higher in patients with severe compared to non-severe disease (**Table 1**). Five out of 12 patients (two non-severe and three severe) who accepted to give blood after six months reported long-term COVID-19.

### Persistent İncrease in Blood Monocytes and Decrease in DC and NK Cell Subsets in COVID-19 Patients

**Figure 1:**
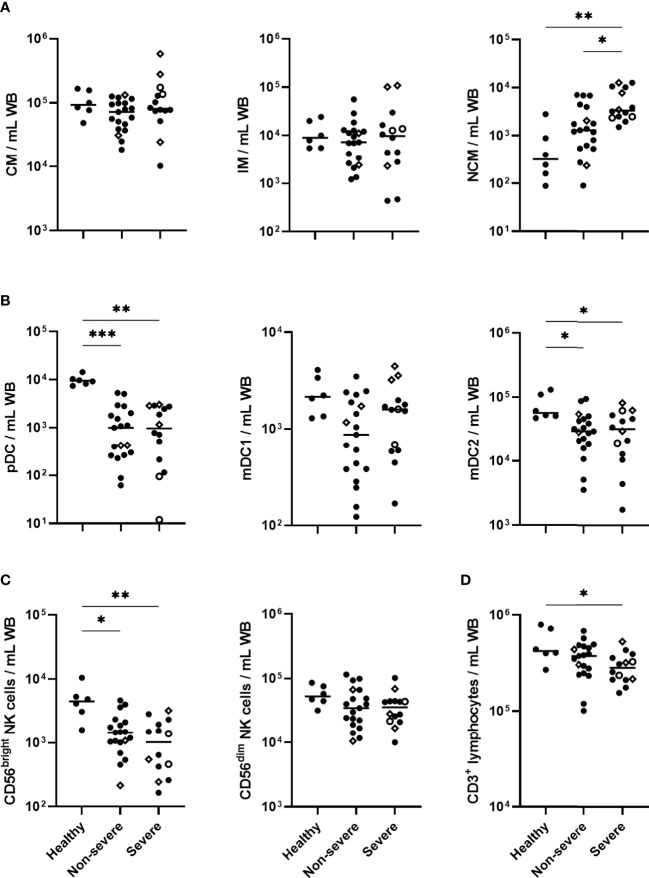
Absolute cell numbers of monocytes, dendritic cell (DC) subsets, NK cell subsets and CD3^+^ lymphocytes in COVID-19 patients compared to healthy controls. Absolute numbers of **(A)** classical monocytes (CM), intermediate monocytes (IM) and non-classical monocytes (NCM); **(B)** plasmacytoid DC (pDC), myeloid DC1 (mDC1) and myeloid DC2 (mDC2); **(C)** CD56^bright^ and CD56^dim^ NK cells **(D)** CD3^+^ lymphocytes, are represented for healthy controls (n=6), non-severe (n=19) and severe (n=14) COVID-19 patients at day 0. The patients who died from COVID-19 are represented on the graphs as empty circles, and the patients who declared long-term COVID-19 at their 6 months follow-up (2 out of 8 non-severe and 3 out of 4 severe cases) are represented on the graphs as empty diamonds. The horizontal bars indicate the medians of the results within each column. The significant *p* values < 0.05 shown on the graphs were obtained by Kruskal Wallis test, and they are indicated as *p<0.05, **p<0.01, ***p<0.001.

Peripheral blood monocytes, dendritic cells, NK cells and CD3^+^ T lymphocytes were characterized by flow cytometry at three different time points. The distribution of absolute numbers of the cell subsets was heterogeneous, with some significant differences between the patient subgroups and controls. On day 0, whereas no significant differences between patients and controls were noticed for classical and intermediate monocytes, higher numbers of non-classical monocytes were observed in severe COVID-19 patients than patients with non-severe forms and controls (**Figure 1A**). The numbers of blood non-classical monocytes remained higher in both non-severe and severe COVID-19 patients compared to controls for at least up to 6 months (**Supplementary Figure 3A**).

In contrast to blood monocytes, a sharp drop in the numbers of pDC and mDC2 subsets, and a trend for mDC1 subset, was observed in COVID-19 patients at day 0 (**Figure 1B**). These cell numbers remained low until six months in non-severe and severe patients compared to healthy controls (**Supplementary Figure 3B**).

A marked decrease in the CD56^bright^ NK cells was noticed among the lymphocytes in COVID-19 patients on day 0. It was more significant in severe forms and was accompanied by lower numbers of CD3^+^ T lymphocytes, consistent with their lymphopenia (**Figures 1C, D**). Compared to controls, the low numbers of blood CD56^bright^ NK cells persisted up to 6 months, whereas the CD3^+^ T lymphopenia had disappeared (**Supplementary Figures 3C, D**). No significant change in blood CD56^dim^ NK cell numbers was observed in COVID-19 patients (**Figure 1C**).

### Persistent Over-Expression of IL-22R1 on Monocyte and DC Subsets in COVID-19 Patients

**Figure 2:**
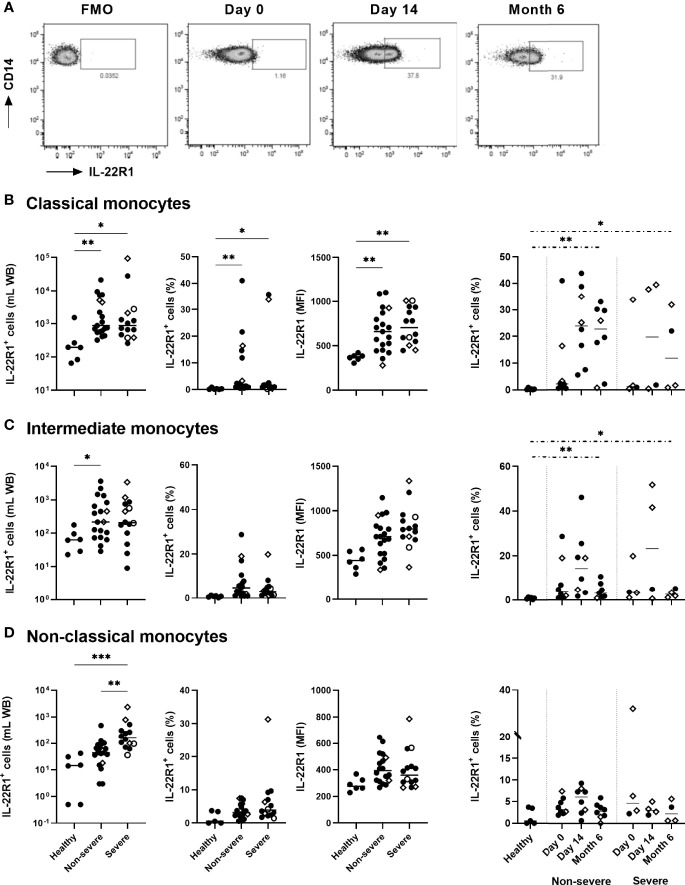
IL-22R1 expression on human blood monocyte subsets in COVID-19 patients compared to healthy controls. **(A)** Representative pseudo-color plots of IL-22R1^+^ expression on classical monocytes from a severe COVID-19 patient, with a Fluorescence Minus One (FMO) control and the expressions at day 0, day 14 and six months later. The rectangular gate is used to identify the distinct upcoming population of myeloid cells expressing higher density of IL-22R1. The presentation of this distinct population became clearer at D14 and remained at least for 6 months. **(B–D)** Absolute cell numbers, percentages of the IL-22R1^+^ cells and median fluorescence intensity of IL-22R1 on **(B)** classical, **(C)** intermediate and **(D)** non-classical monocytes are shown for healthy controls (n=6), non-severe (n=19) and severe (n=14) COVID-19 patients at day 0. Kinetic analysis of the percentages of IL22R1^+^ cells over time is represented for COVID-19 patients on the right panels at D0 and 14 days (11 non-severe, 9 severe) and six months (8 non-severe, 4 severe) later. The patients who died from COVID-19 are represented on the graphs as empty circles, and the patients who declared long-term COVID-19 at their 6 months follow-up (2 out of 8 non-severe and 3 out of 4 severe cases) are represented on the graphs as empty diamonds. The horizontal bars indicate the medians of the results within each column. The statistical analyses were performed by Kruskal Wallis test to compare results from the different groups of subjects at D0, and by Mann Whitney U test to compare patient’s results at Month 6 to controls. Significant differences are shown as *p<0.05, **p<0.01, ***p<0.001. Time point comparisons at D0, D14 and Month 6 restricted to paired samples were performed by Friedman test and no significant differences were noticed.

The *ex vivo* expression of IL-22R1 on monocytes and DC subsets was evaluated on whole blood by flow cytometry as absolute numbers and percentages of IL-22R^+^ expressing cells and as MFI of the receptor on the myeloid cells. As illustrated in **Figure 2A** with a representative IL-22R1 expression on classical monocytes from a severe COVID-19 patient, the fluorescence density of IL-22R1 on monocyte subsets was higher than the fluorescence minus one (FMO). It was distributed over the entire cell population at the beginning of infection (Day 0). In addition, an increase in the proportions of a distinct subset of IL-22R1^+^ cells, characterized by an even higher fluorescence density of IL-22R1, became prominent at day 14 and persisted at least until six months (**Figure 2A**).

The numbers of IL-22R1^+^ classical, intermediate, and non-classical monocytes were higher in patients than controls at day 0, whereas the percentages and the density of IL-22R1 expression among each cell subset reached statistically significant differences only for classical monocytes. However, the most significant rise in the numbers of IL-22R1^+^ monocyte subset was noticed for non-classical monocytes in severe patients, as a consequence of the high numbers of non-classical monocytes in these patients. Higher numbers of IL-22R1^+^ non-classical monocytes were thus observed at day 0 in severe compared to non-severe patients.

The proportions of IL-22R1^+^ classical and intermediate monocytes remained high for six months in the two groups of patients, compared to controls (**Figures 2B–D**). In contrast, a progressive decrease was observed in the density of IL-22R1 on the three monocyte subsets (data not shown). The figure from a representative sample of a severe COVID-19 patient shows the decrease of the density of IL-22R1 expression on classical monocytes from D14 to M6 (**Figure 2A**).

**Figure 3:**
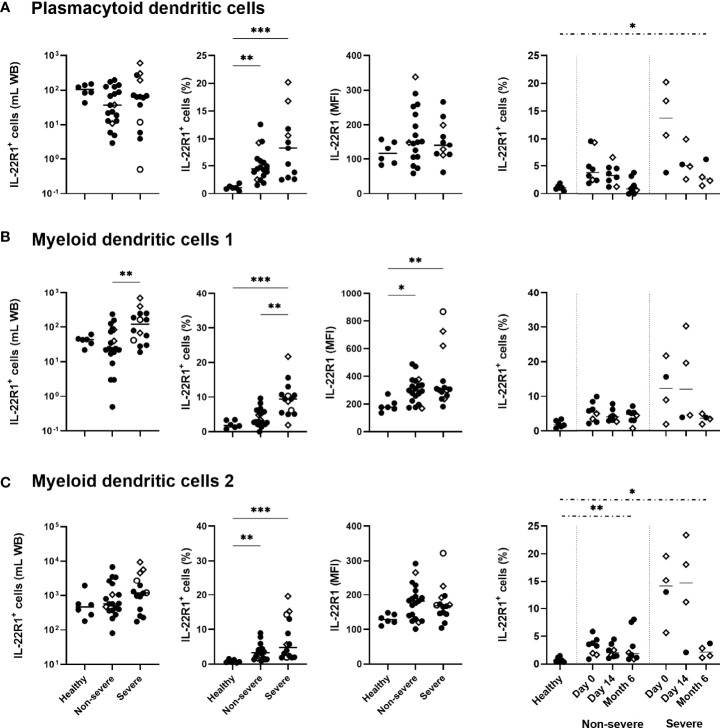
IL-22R1 expression on human blood dendritic cell (DC) subsets in COVID-19 patients compared to healthy controls. Absolute cell numbers, percentages of the IL-22R1^+^ cells and median fluorescence intensity of IL-22R1 on **(A)** plasmacytoid DC, **(B)** myeloid DC1 and **(C)** myeloid DC2 are shown in healthy controls (n=6), non-severe (n=19) and severe (n=14) COVID-19 patients at day 0. Kinetic analysis of the percentages of IL22R1^+^ cells over time is represented for COVID-19 patients on the right panels at D0 and 14 days (11 non-severe, 9 severe) and six months (8 non-severe, 4 severe) later. The patients who died from COVID-19 are represented on the graphs as empty circles, and the patients who declared long-term COVID-19 at their 6 months follow-up (2 out of 8 non-severe and 3 out of 4 severe cases) are represented on the graphs as empty diamonds. The horizontal bars indicate the medians of the results within each column. The statistical analyses were performed by Kruskal Wallis test to compare results from the different groups of subjects at D0, and by Mann Whitney U test to compare patient’s results at Month 6 to controls. Significant differences are shown as *p<0.05, **p<0.01, ***p<0.001. Time point comparisons at D0, D14 and Month 6 restricted to paired samples were performed by Friedman test and the numbers of IL-22R1^+^ pDC cells were lower at 6 months compared to D0 in non-severe patients (*p*<0.01).

Regarding DC, higher percentages of IL-22R1^+^ pDC, mDC1 and mDC2 were found in COVID-19 patients than controls, which was associated with a higher density of this receptor on mDC1. Severe COVID-19 patients were characterized by a trend for higher percentages of IL-22R1^+^ pDC and mDC2 cells and by significantly higher proportions of IL-22R1^+^ mDC1 cells than non-severe patients (**Figures 3A–C**). Similarly to their percentages, absolute numbers of IL-22R1^+^ mDC1 cells were higher in severe COVID-19 patients compared to non-severe patients. In contrast, no difference was noticed between patients and controls for absolute numbers of IL-22R1^+^ pDC and mDC2 cells, in spite of higher proportions of these cells, probably due to the diminution of the total numbers of pDC and mDC2 in patients (**Figures 3A–C**).

The percentages of IL-22R1^+^ DC progressively decreased over time. However, the proportions of IL-22R1^+^ pDC and mDC2 cells remained abnormally high in patients at six months compared to controls, in severe patients for pDC and mDC2 and in non-severe patients for mDC2 (**Figures 3A–C**).

### Discriminative Value of IL-22R1 Expression on Myeloid Cells for the Severity of COVID-19

**Figure 4:**
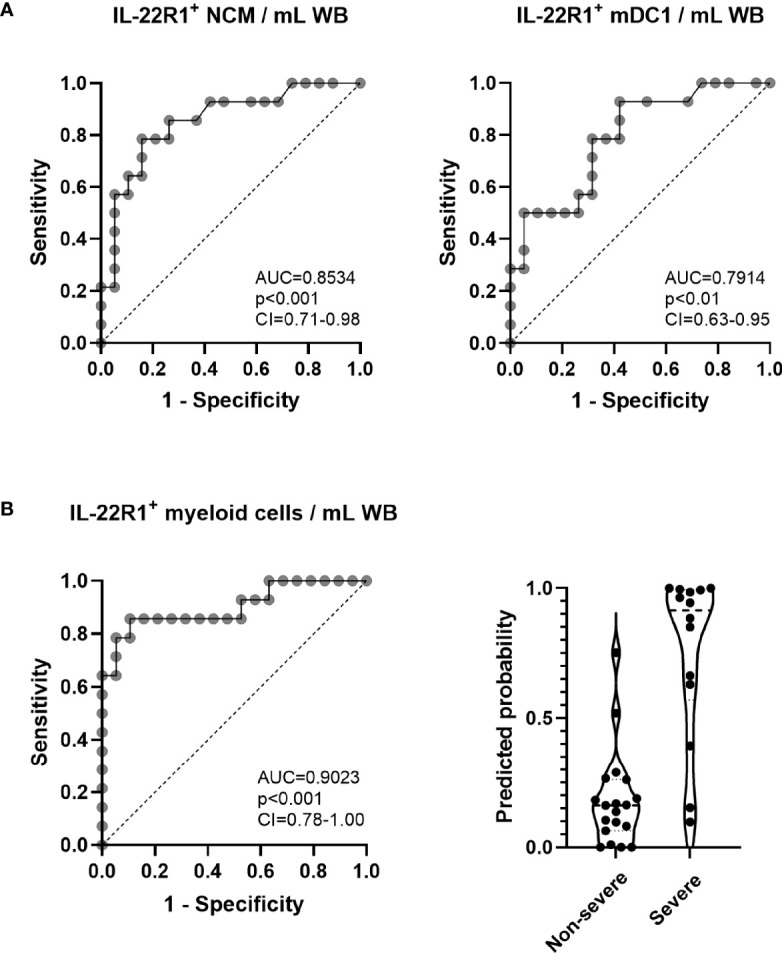
Receiver operating characteristic (ROC) curves and logistic regression analysis of IL-22R1^+^ myeloid cells to discriminate severe from non-severe COVID-19 patients. **(A)** ROC curves of IL-22R1^+^ non-classical monocytes (NCM) and myeloid DC1 (mDC1) were performed to compare severe from non-severe COVID-19 patients. The dotted lines represent an AUC of 0.5. **(B)** ROC curve of IL22R1+ myeloid cells and the predicted probabilities of logistic regression model in non-severe (n=19) and severe (n=14) COVID-19 patients. The dotted line shown on the ROC curve represents an AUC of 0.5. The dotted horizontal lines on panel **(B)** (right part) represent the medians of the linear prediction values. AUC, area under the ROC curve; CI, confidence interval; WB, whole blood.

Considering the numbers of IL-22R1^+^ myeloid cells, three monocyte subsets and three DC subsets, which were higher for some cell subsets in patients with severe than non-severe disease, we performed a univariate and multivariate logistic regression analysis, and we depicted receiver operating characteristic (ROC) curves for non-severe vs severe COVID‐19 disease. Results indicated significant differences for IL-22R1^+^ non-classical monocytes and mDC1 for discriminating non-severe diseases from severe ones (AUC=0.8534 and 0.7914 respectively, **Figure 4A**).

Moreover, to define a more robust biomarker of severity, we combined the results obtained for the numbers of the six subsets of IL-22R1^+^ myeloid cells by multivariate regression analysis. These results provided further valuable discrimination according to the severity of the disease (AUC=0.9023, **Figure 4B**). The predicted probability of presenting a severe versus a non-severe disease based on this multivariate analysis of IL-22R1^+^ myeloid cells is represented in **Figure 4B** (right panel).

### Activation of IL-22R^+^ Myeloid Cells in COVID-19 Patients

**Figure 5:**
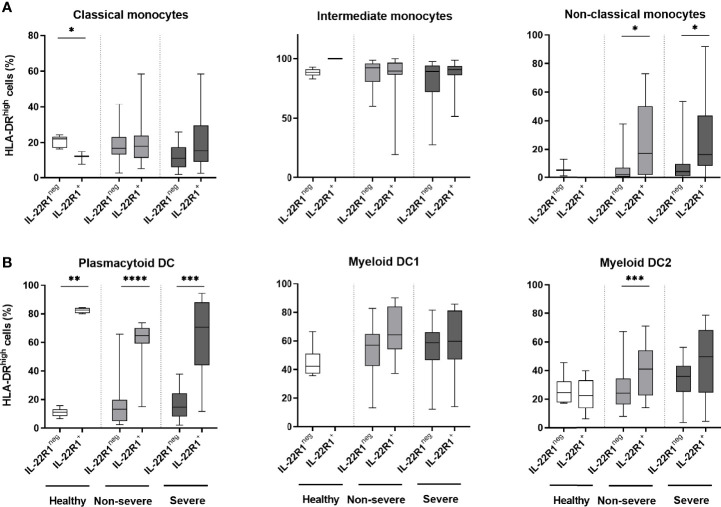
HLA-DR^high^ expression on IL-22R1^+^ and IL-22R1^-^ myeloid cell subsets in COVID-19 patients and controls. The percentages of HLA-DR^high^ expression on IL-22R1^+^ and IL-22R1^-^ cells at Day 0 are represented for **(A)** monocyte subsets and **(B)** DC subsets, in healthy controls (n=6), non-severe (n=19) and severe (n=14) COVID-19 patients. The boxes show the 25-75% interquartile ranges, horizontal bars indicate the medians and vertical bars indicate the minimum and maximum of the results within each column. The significant *p* values are indicated on the graphs as *p<0.05, **p<0.01, ***p<0.001 and ****p<0.0001 (Mann Whitney U test).

HLA-DR expression has been described as an activation marker of the myeloid cells (33, 37). To evaluate the activation status of IL-22R1 expressing myeloid cells, we next assessed their HLA-DR expression patterns in SARS-CoV-2 infection. The expression of HLA-DR^high^ was not higher on IL-22R1^+^ classical and intermediate monocytes compared to their IL-22R1^neg^ counterparts (**Figure 5A**). In contrast, the percentages of HLA-DR^high^ was higher among IL-22R1^+^ non-classical monocytes than their IL-22R1^neg^ counterparts both for non-severe and severe patients, but not for controls (**Figure 5A**), indicating a disease-related activation of IL-22R1^+^ non-classical monocytes.

Patients with COVID-19 also had higher expression of HLA-DR^high^ on IL-22R1^+^ pDC compared to their IL-22R1^neg^ cells, but this higher expression was also observed in controls suggesting that this IL-22R1 related activation pattern is not restricted to SARS-CoV-2 infection. Higher HLA-DR expression on IL-22R1 expressing mDC2 than IL-22R1^neg^ mDC2 was also noticed in COVID-19 patients, mostly in non-severe ones (**Figure 5B**).

### Plasma Pro-Inflammatory Cytokines and Chemokines and Their Association With IL-22R1 Expressing Myeloid Cells

We measured 16 cytokines/chemokines in the plasma collected from 6 controls, 10 non-severe and 11 severe patients at day 0. The concentrations of seven of them (GM-CSF, IL-7, IL-8, IL-17A, IL-22, MIP-1α, and MIP-1β) were not detectable or did not show any differences between controls and COVID-19 patients, and were thus not further analyzed. In contrast, we found that plasma IL-6, IFN-α, IFN-γ, IL-1β, TNF-α, IL-10, IP-10, MIG and MCP-1 levels were significantly higher in severe COVID-19 compared to healthy controls. IFN-α and TNF-α concentrations were also significantly higher in non-severe COVID-19 compared to healthy controls (**Supplementary Table 2**).

**Figure 6:**
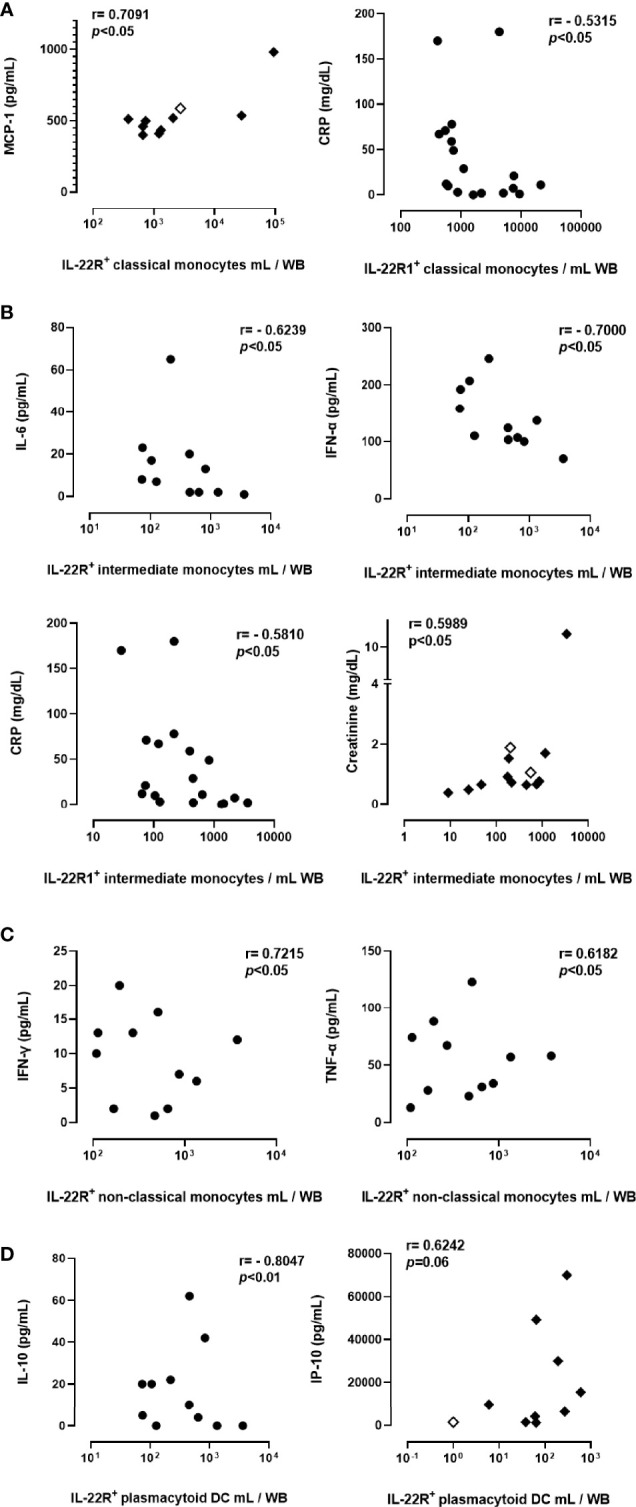
Correlations between the numbers of IL-22R1^+^ myeloid cell subsets and blood parameters in COVID-19 patients. Only the significant results are represented on the figure for correlations of IL-22R1 expressing classical monocytes **(A)**, intermediate monocytes **(B)**, non-classical monocytes **(C)** and plasmacytoid DC **(D)** with plasma pro-inflammatory mediators. Circles and filled diamonds represent non-severe and severe COVID-19 patients, respectively. The empty diamonds were used for patients who died from COVID-19. R and p values obtained by non-parametric Spearman’s test are indicated on the panels.

Correlation analysis between the numbers of IL-22R1^+^ myeloid cells subsets and plasma pro-inflammatory cytokines and chemokines of interest, as well as blood parameters reported to be associated with disease severity, revealed several correlations. The numbers of IL-22R1^+^ classical monocytes were positively correlated with plasma MCP levels in severe COVID-19 (r=0.7091, *p*<0.05) and negatively correlated with CRP concentrations (r=-0.5315, *p*<0.05) in patients with the non-severe disease (**Figure 6A**). The numbers of IL-22R1^+^ intermediate monocytes were negatively correlated with IL-6 (r=-0.6239, *p*<0.05), IFN-α (r=-0.7000, *p*<0.05) and CRP concentrations (r=0.5810, *p*<0.05) in non-severe infections, whereas they were positively correlated with creatinine levels (r=0.5989, *p*<0.05*)* in severe cases (**Figure 6B**). IL-22R1^+^ non-classical monocytes were positively correlated with IFN-γ (r=0.7215, *p*<0.05) and TNF-α (r=-0.6182, *p*<0.05) in non-severe infections (**Figure 6C**). Finally, IL-22R1^+^ pDC were negatively correlated with IL-10 concentrations in non-severe cases (r=-0.8041, *p*<0.01), but positively correlated with IP-10 levels in severe infections (r=0.6242, *p*=0.06) (**Figure 6D**). No correlation was found between IL-22R1^+^ monocytes or DC subsets and neutrophils or lymphocytes numbers or neutrophil/lymphocyte ratio.

### Cytokine and Cytotoxicity Mediator Expressions of NK Cells and T Lymphocytes in COVID-19 Patients

**Figure 7:**
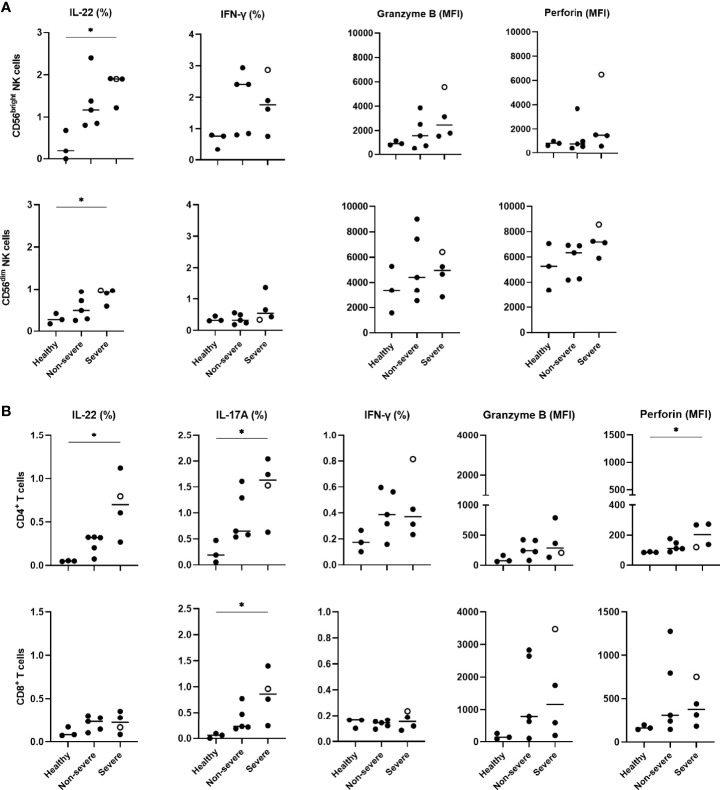
Cytokine expressions and cytotoxicity mediators of peripheral blood NK and T cells in COVID-19 patients compared to healthy controls. **(A)** IL-22, IFN-γ, granzyme B and perforin expressions on CD56^bright^ and CD56^dim^ NK cells, **(B)** IL-22, IL-17A, IFN-γ, granzyme B and perforin expressions on CD4^+^ and CD8^+^ lymphocytes, are shown according to study groups (3 healthy controls, 5 non-severe and 4 severe patients). PBMC were left unstimulated in supplemented RPMI medium during 24 hours before staining them with the different monoclonal antibodies to identify the IL-22, IL-17A, IFN-γ, granzyme B and perforin content of CD56^bright^ and CD56^dim^ NK cells, CD4^+^ and CD8^+^ lymphocytes. Results of cytokine content are indicated as percentages of cytokine-containing cells whereas those of granzyme B and perforin are indicated as the MFI of the subset-containing cells. The patient who died from COVID-19 is represented by an empty circle. The horizontal bars indicate the medians of the results within each column. Non-paired data were analyzed by the Kruskal Wallis test, and the significant *p* values are indicated as *p<0.05.

To explore a possible relationship between IL-22R1 expression by myeloid cells and the functional activity of NK cells or T lymphocytes in COVID-19 patients, the percentages of IL-22, IL-17 and IFN-γ-containing lymphocytes as well as the density of expression for granzyme B and perforin was analyzed by flow cytometry on PBMC, both in the absence and presence of *in vitro* stimulation. The frequencies of IL-22^+^ CD56^bright^ and CD56^dim^ NK cells detected in the absence of *in vitro* stimulation were higher in COVID-19 patients than controls. Significant differences were observed in severe patients (**Figure 7A**). A trend for higher frequencies of IFN-γ^+^ CD56^bright^ was noticed in parallel, and trends for higher MFI for granzyme B and perforin mostly on CD56^dim^ NK cells (**Figure 7A**). In contrast, IL-22 and IFN-γ responses of NK cells to *in vitro* stimulation were reduced in most COVID-19 patients compared to controls. Still, the cytotoxic mediator expressions were similar in patients and controls (**Supplementary Figure 4A**).

Whereas no significant or very low percentages of *ex vivo* IL-22 or IL-17A-containing T lymphocytes were observed in healthy controls, IL-22^+^ and IL-17A^+^ single positive CD4^+^ and IL-17A^+^ CD8^+^ T cells were detected in COVID-19 patients. The percentages of these cells were significantly higher in severe patients compared to healthy subjects, albeit they were abnormal even in non-severe patients (**Figure 7B**). Trends for higher proportions of IFN-γ-containing CD4^+^ T lymphocytes and higher granzyme B MFI were observed in COVID-19 patients. A significant increase in the density of perforin on these cells was also noticed in severe COVID-19 patients (**Figure 7B**). Similar to NK cells, the frequencies of IFN-γ-containing CD4^+^ T cells in response to *in vitro* stimulation was low in severe COVID-19 patients compared to controls. In contrast, a further increase in the percentages of IL-22^+^ CD4^+^ T lymphocytes was noticed after stimulation with a similar rise in the granzyme B and perforin density both in patients and controls (**Supplementary Figure 4B**). Additionally, the Boolean analysis indicated that IL-22^+^ NK cells did not co-express IFN-γ, granzyme B or perforin and that IL-22 and/or IL-17A expressing T cells also did not co-express IFN-γ, granzyme B and perforin (data not shown).

A correlation matrix for IL-22R^+^ expressing myeloid cell subsets with the *ex vivo* IL-22, IL-17A, IFN-γ, granzyme B and perforin expressions on NK and T cells was generated to evaluate a possible relationship between these parameters in COVID-19 patients. The percentages of IFN-γ^+^ CD56^dim^ NK cells were negatively correlated with the numbers of IL-22R1^+^ classical monocytes (r=0.6833, *p*<0.05), whereas the frequency of IL-22 producing CD56^dim^ NK cells was positively correlated with the numbers of IL-22R1^+^ non-classical monocytes (r=0.7167, *p*<0.05), (data not shown).

### Distinct Patterns of IL-22R1 Expression on CD4^+^ T Lymphocytes Defined in COVID-19 Patients

**Figure 8:**
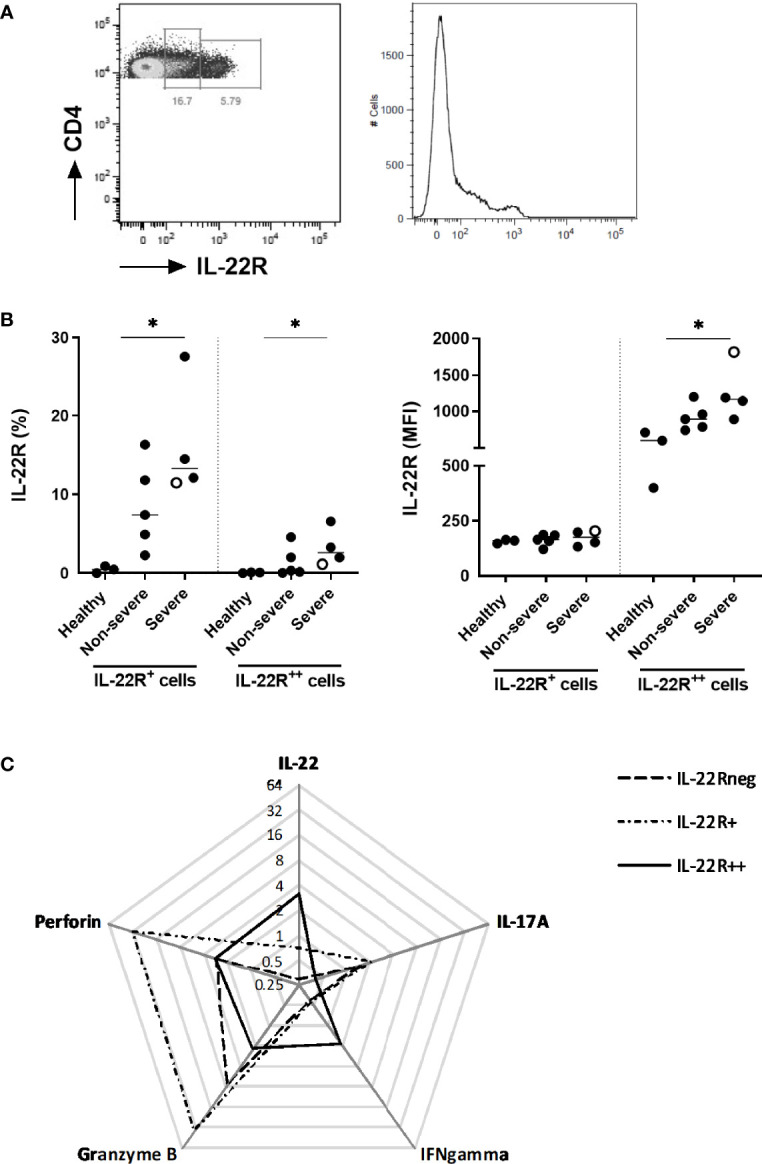
Distinctive IL-22R1 expression patterns of peripheral blood CD4^+^ lymphocytes. **(A)** Representative pseudo-color plot and histogram showing IL-22R^+^ and IL-22R^++^ expression on CD4^+^ T lymphocytes from a representative severe COVID-19 patient. **(B)** Percentages and median fluorescence intensity of IL-22R1^+^ and IL-22R1^++^ CD4^+^ T cells on the day of inclusion for healthy controls (n=3), non-severe (n=5) and severe patients (n=4). The patient who died from COVID-19 is represented as an empty circle. The horizontal bars indicate the medians of the results within each column. **(C)** Radar chart of the median frequencies of IL-22, IL-17A, IFN-γ, granzyme B and perforin expressions are represented for IL-22R1^neg^, IL-22R1^+^ and IL-22R1^++^ CD4^+^ T cells. The significant p values obtained by the Kruskal Wallis test, are indicated as *p<0.05.

Considering that the numbers of IL-22^+^ CD4^+^ T cells were increased in COVID-19 patients and that IL-22R1-expressing cells may induce a positive auto-regulatory loop with IL-22 expression (20), the IL-22R1 expression was investigated on PBMC in parallel to the functional evaluation of the CD4^+^ lymphocytes. Two different subsets of CD4^+^ cells were defined as IL-22R1^+^ and IL-22R1^++^ according to their MFI and patterns of IL-22R1 expression (**Figure 8A**). The percentages of IL-22R1^+^ and IL-22R1^++^ CD4+ T lymphocytes were significantly higher in severe patients than controls, and the MFI of IL-22R was also clearly higher in severe diseases (**Figure 8B**). The percentages of both IL-22R1^+^ and IL-22R1^++^ CD4^+^ T lymphocytes were positively correlated with the frequencies of IL-22-expressing CD4^+^ T cells (r=0.7622, *p*<0.01 and r=0.6573, *p*<0.05, respectively).

These two patterns of IL-22R1 expressions of CD4^+^ T lymphocytes delineated functionally different subsets. IL-22R^+^ CD4^+^ T cells were characterized by higher proportions of cells containing granzyme B, perforin and IL-17A, whereas the IL-22R^++^ CD4^+^ T cells had higher proportions of IL-22 and IFN-γ compared to IL-22R^+^ CD4^+^ T cells (**Figure 8C**).

### Plasma IL-22 and IL-22-BP Concentrations of COVID-19 Patients

**Figure 9:**
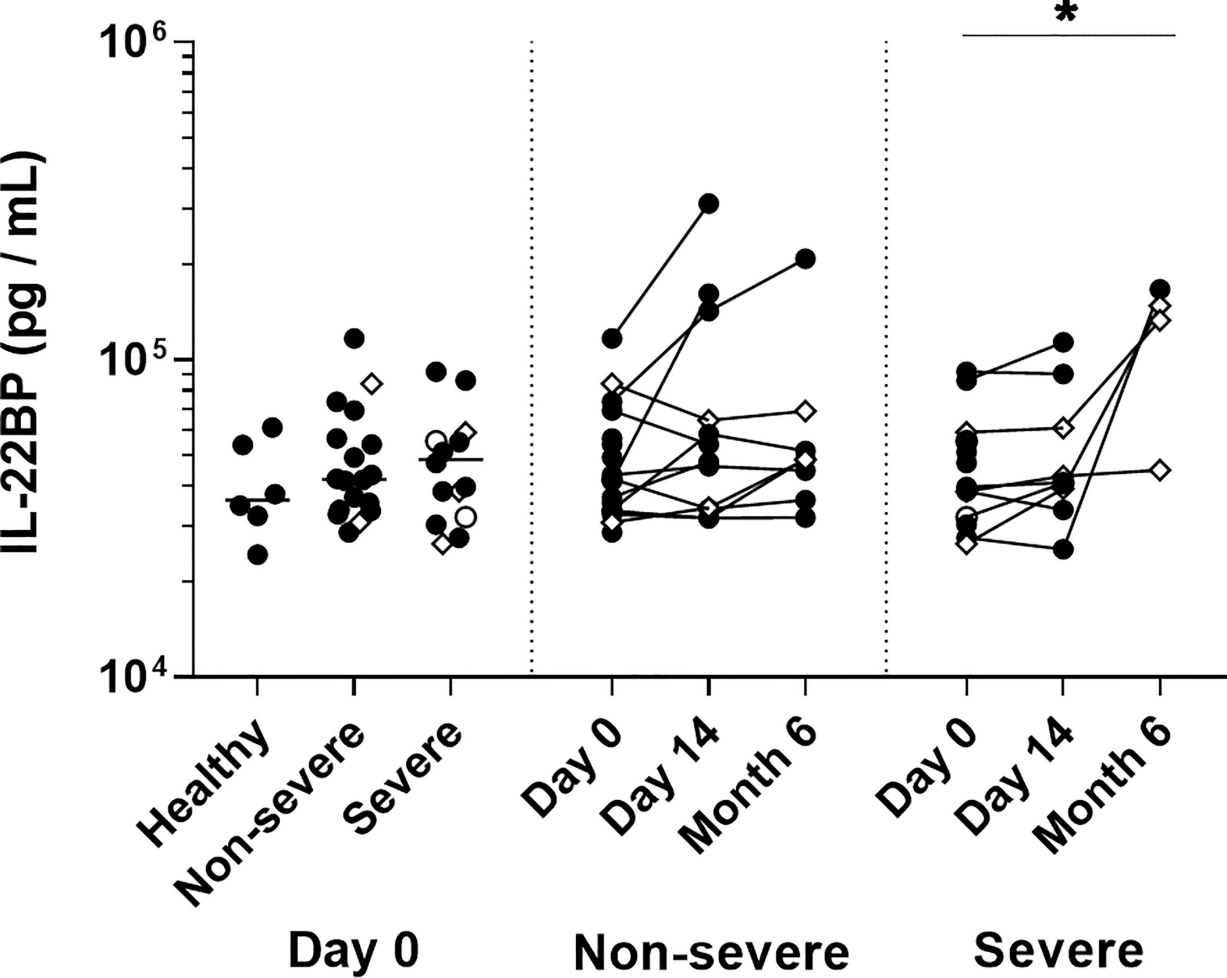
Plasma IL-22 binding protein levels in COVID-19 patients. IL-22BP levels measured on the day of admission are represented on the left panel for healthy controls (n=6), non-severe (n=18) and severe (n=14) COVID-19 patients. The horizontal bars indicate the medians of the results within each column at D0. Kinetic analysis of IL-22BP levels over time is represented for 11 non-severe and nine severe COVID-19 patients at D0 and D14, and for eight non-severe and four severe patients six months after their inclusion The patients who died from COVID-19 are represented as an empty circle, and the patients who declared long-term COVID-19 at their 6 months follow-up (2 out of 8 non-severe and 3 out of 4 severe cases) are represented on the graphs as empty diamonds. The significant *p* values < 0.05 is represented on the graph as *, obtained both by Kruskal Wallis test and Friedman U test.

IL-22R1 is the natural ligand for IL-22, and the binding of IL-22 to its membrane receptor is tightly regulated by soluble IL-22BP (15). In this study, we measured plasma IL-22 and IL-22BP concentrations by ELISA. IL-22 was detectable neither in plasmas of COVID-19 patients nor in healthy controls. In contrast, IL-22BP was detectable in all plasma samples, but no difference was found between patients and controls at day 0. At six months, the plasma levels of IL-22BP had risen only in a few patients compared to their day 0 levels, mainly among the severe ones (**Figure 9**). The plasma IL-22BP concentrations were not correlated with the clinical data or increasing numbers of IL-22R1^+^ monocytes or DC subsets.

## Discussion

Due to an extended pro-inflammatory state, SARS-CoV-2 infection leads to various clinical spectra from mild to severe clinical manifestations (2, 11, 38). This study investigated the IL-22/IL-22R axis in SARS-CoV-2 infection, defined either as a protective or a harmful pathway in different diseases with excessive inflammatory conditions (16–20). We focused on the dynamics of the immune cells in the blood in parallel to the alterations of IL-22R1 expression on these cells in non-severe and severe COVID-19 patients without any corticosteroid treatment as they were recruited during the first wave of the pandemic. We observed a sharp drop of blood DC subsets and a significant increase in non-classical monocytes in line with previously reported data (39). In contrast, others reported a decrease in non-classical monocytes (2). These discrepancies could be due to different gating strategies of the cells or different patients selections. More importantly, we report an over-expression of IL-22R1 on the different peripheral blood myeloid cell subsets as a hallmark of SARS-CoV-2 infection, with a particular expression pattern of the IL-22R1 on monocytes, starting with a global increase in the density on entire cell subsets, followed by an increase of the numbers of IL-22R1 expressing cells. This over-expression on myeloid cells provided a high predictive value between severe and non-severe disease. In addition, high numbers of different subsets of IL-22R1 expressing myeloid cells were correlated with the plasma concentrations of various immune mediators described as important in the pathogenesis of SARS-CoV-2 infection, strengthening the potential importance of IL-22/IL-22R1 axis in COVID-19.

SARS-CoV-2 enters the human body *via* the respiratory tract epithelial cells and migrates to the lung, triggering local immune responses (38). So far, the expression of IL-22R1 on airway epithelial cells has not been investigated in COVID-19, whereas it is expressed on these cells in normal physiological conditions. Its expression has been reported to be increased in pathological conditions such as influenza infection both in animal models and humans, where it mediates epithelial cells repair in the lung (13, 14, 24, 40, 41). A similar mechanism might be involved during SARS-CoV-2 infection. Still, due to the difficulties of sampling airway epithelial cells in humans, we could not investigate the involvement of this receptor directly in lung cells and focused on blood myeloid cells. When COVID-19 disease progresses, several abnormal immune responses occur, leading to systemic features related to the severity of the disease. The severe forms are marked by distributional and functional changes of the immune cells, leading to a generalized hyperinflammatory state in peripheral blood cells that could reflect the lung inflammatory status (4, 7, 10–12, 40, 42, 43). It has indeed been shown that cytokine secretion in the lung drives the systemic redistribution of immune cells in peripheral blood of COVID-19 patients (40). We show here that this redistribution is associated with an abnormal expression of IL-22R1 on different subsets of blood myeloid cells. Even if IL-22R1 usually is not expressed on blood myeloid cells, its expression was reported to be induced during infections and/or inflammatory conditions, but the reasons for this abnormal expression remain unclear (16, 19, 20).

In patients with severe COVID-19, we found a positive correlation between IL-22R1^+^ classical monocytes and plasma MCP-1 levels, suggesting recruitment-related increase on these cells. The expression of IL-22R1 was reported on macrophages within the granulomas during the chronic stage of *Mycobacterium tuberculosis* infection, which was linked to the recruitment of the infected macrophages to the lung and the control process of the bacteria (19, 20, 44). Similarly, IL-22R signaling pathway activation after viral infections, like Hepatitis B Virus (HBV) in the liver and West Nile Virus in the central nervous system, has been shown as responsible for chemokine mediated recruitment to the infection site, representing a beneficial role for the former, but inducing pathological tissue damage for the latter (14). Given the immune cell recruitment-related pleiotropic effect of IL-22R1 against different microorganisms in different tissues (14), it is difficult, without further functional analysis, to assume whether these recruitment-related changes in IL-22R1 expression in SARS-CoV-2 infection is beneficial for the body or not. However, the positive correlations found in COVID-19 patients between IL-22R1^+^ classical monocytes and plasma MCP-1 concentrations and between IL-22R1^+^ pDC and the plasma IP-10 levels in patients with a severe disease suggest a deleterious effect of IL-22R1 expressing cells as both MCP-1 and IP-10 levels are associated with the severity of the disease and pathological lung inflammation (14, 45). Non-severe patients were characterized by positive correlations between numbers of IL-22R1^+^ non-classical monocytes and plasmas TNF-α and IFN-γ concentrations. These different parameters probably reflect the high activation of immune cells. Negative correlation between IL-22R1 pDC and IL-10 concentrations in non-severe patients might be related to the anti-inflammatory properties of IL-10 (46). In contrast, negative correlations between IL-22R1^+^ classical and intermediate monocytes and CRP levels, and IL-22R1^+^ intermediate monocytes and plasma IL-6 and IFN-α in non-severe patients suggest a beneficial role of IL-22R1 expressing cells in COVID-19 as CRP, IL-6, and IFN-α are predictors of lung injury and indicate the severity of SARS-CoV-2 infection (47, 48). These differences between non-severe and severe patients could indicate a differential role of IL-22R1 expression on myeloid cells at different stages of the infection. This expression could be beneficial in the beginning and, in contrast, detrimental when the inflammatory responses progress in severe patients. Different expression patterns in various subsets, and the diversity of the correlations with the pro-inflammatory mediators suggested that the activation of IL-22R1 expression on myeloid cell subsets are related to the cell subsets *per se* and the inflammatory milieu.

To better understand the significance of IL-22R1 expression on myeloid cells in SARS-CoV-2 infection, we analyzed the HLA-DR expression of IL-22R1^+^ cells, as HLA-DR expression describes the activation status of antigen-presenting cells (49, 50). Low expression of HLA-DR is associated with dysfunctionality and immunoparalysis of monocytes at the early phase of SARS-CoV-2 infection, whereas mild disease has been characterized by HLA-DR^high^ monocytes (8, 37). We report higher expression of HLA-DR^high^ on IL-22R1^+^ mDC2 from non-severe patients and IL-22R1^+^ non-classical monocytes from both non-severe and severe patients compared to their IL-22R1^neg^ counterparts. As these two cell subsets are essential antigen-presenting cells during viral infections (33–35), the higher HLA-DR^high^ expression by IL-22R1^+^ cells suggests that IL-22R1 expression might be a compensatory mechanism of activation of these cells to enhance their antigen-presenting capacity. This might be especially important for IL-22R1^+^ non-classical monocytes as their numbers are seriously increased at the beginning of the SARS-CoV-2 infection. This high IL-22R1^+^ HLA-DR^high^ co-expression could result from a direct effect of viral antigens (51), as is the case on airway epithelial cells (13), and could be involved in viral antigens presentation to lymphocytes.

As a result of higher antigen presentation capacities probably associated with IL-22R1 expression, different subsets of CD4^+^ lymphocytes from severe patients were quite activated as, in the absence of any *in vitro* stimulation, they produced IL-22 and IL-17A, and to a lower extent IFN-γ, associated to increased expression of perforin. Subsets of IL-17-producing CD8^+^ T cells and IL-22-producing NK cells were activated in parallel. IL-22-producing CD56^dim^ NK cells, a subset with a relatively stable number of cells in COVID-19 patients, were positively correlated with the numbers of IL-22R1^+^ non-classical monocytes, the subset of IL-22R1^+^ monocytes with the highest rise in severe COVID-19 patients. These NK cells probably provide the ligand to the receptor expressed on non-classical monocytes leading to their activation. The IL-22/IL-22R pathway is thus involved in innate immune responses occurring during SARS-CoV-2 infection. This pathway was also reported to provide innate protective immunity in the absence of adaptive immune response during infection with human immunodeficiency virus (24). The high *in vivo* activation of both NK cells and T lymphocytes probably explains their hypo-responsiveness to an *in vitro* stimulation. This is in line with the reduced IFN-γ secretion and antiviral cytotoxic responses of lymphocytes reported for severe COVID-19 patients as a consequence of their exhaustion (52–55). Such exhaustion of IFN-γ-secreting CD56^dim^ NK cells could explain their negative correlation with the numbers of IL-22R1^+^ classical monocytes as we hypothesized that SARS-CoV-2-infected classical monocytes express IL-22R1 as a compensatory activation mechanism to their low activation by IFN-γ (51).

Like for other viral infections, HIV-1 and HBV, we demonstrated that in addition to innate immune responses, the cells of adaptative immune responses are involved in IL-22 production during SARS-CoV-2 infection (14). IL-22-producing CD4^+^ T lymphocytes also expressed IL-22R1, and based on the intensity of expression of this receptor, we identified two different subsets of IL-22R1 expressing CD4^+^ T lymphocytes IL-22R1^+^ and IL-22R1^++^. Both subsets contained IL-22-producing CD4^+^ T lymphocytes. However, whereas IL-22R1^+^ subset comprised mostly IL-17A, granzyme B and perforin-producing cells, IL-22R1^++^ subset comprised mostly IL-22 and IFN-γ-producing cells, suggesting different roles for IL-22R1 expressing CD4^+^ T cells. The percentage of IL-22R1^+^ CD4^+^ T lymphocytes, both IL-22R^+^ and IL-22R1^++^, was mainly higher in severe patients compared to controls, suggesting a possible compensatory mechanism to impaired CD4^+^ T cell function in COVID-19 patients (52, 55). Interestingly, the severe patient with a fatal outcome had a low percentage of IL-22R1^++^ cells. We hypothesized that this compensatory enlargement of the IL-22R1^++^ CD4^+^ subset might be related to the patient’s survival, but this should be further explored in larger cohorts of patients. We provide thus in this study several arguments suggesting a beneficial role of IL-22R1 expression on immune cells in SARS-CoV-2, even if some results obtained in severe patients suggest an association with the severity of the disease. Similarly, the activation of the IL-22/IL-22R1 cascade reported in different chronic settings was involved in immune protection but overlapped with hyper-inflammatory conditions and even cancers (16, 28, 41). Therefore, it remains challenging to understand the underlying mechanism leading to different outcomes after activation of the IL-22R1/IL-22 axis and this aspect was not investigated in this study. In our cohort, five over 12 patients followed after six months of the initial infection declared to suffer from long-term COVID. However as only 12 patients over the 33 initially included presented after six months, no conclusion could be drawn on the possible impact of IL-22R1 expression on the development of long-term COVID.

The persistent expression of IL-22R1 on classical and intermediate monocyte subsets six months after the initial SARS-CoV-2 infection remains troublesome as we cannot exclude at least in some patients potentially late harmful effects of this receptor expression. However, the possible detrimental interaction of IL-22 with IL-22R is tightly regulated by the naturally occurring antagonist IL-22BP, preventing IL-22 from binding to IL-22R1 and so from tissue damage (15, 24, 28). We did not observe an increase of IL-22BP plasma levels in COVID-19 patients at the early phase of infection, but well six months later for some non-severe and severe patients. This late increase of IL-22BP concentrations could counterpart the high numbers of IL-22R1^+^ classical and intermediate monocytes persisting in some patients. In contrast, for patients with persisting high IL-22R1 expression with no compensatory IL-22BP secretion, we hypothesize that this feature could be associated with a poor prognosis. IL-22 and IL-22R1 were indeed reported to be over-expressed in different types of cancers. They have been related to poor prognosis at later stages of the disease, with low levels of IL-22BP being associated with shorter survival times (28, 56, 57). Future long-term studies of patients who presented COVID-19 should include analysis of persistent membrane expression of IL-22R1 and plasma concentrations of IL-22BP as they could represent predictors of persistent inflammation and/or malignancy development.

In conclusion, our study provides the first evidence that SARS-CoV-2 infection is characterized by the abnormal expression of IL22R1 on blood myeloid cells and CD4^+^ T lymphocytes, the former being a valuable marker to discriminate disease severity. The involvement of this receptor with other cytokines, chemokines and cytotoxic mediators and its co-expression with HLA-DR^high^ suggest a complex mechanism that could be protective and compensatory at the beginning of the infection but could become deleterious at later stages. It is crucial to analyze further the functional role of IL-22/IL-22R1 interactions on immune cells during the recovery phase from COVID-19, as it could shift from a beneficial to a detrimental response over time.

## Data Availability Statement

The raw data supporting the conclusions of this article will be made available by the authors, without undue reservation.

## Ethics Statement

The studies involving human participants were reviewed and approved by “Comité d’Ethique hospitalo-facultaire ERASME-ULB” (021/406). The patients/participants provided their written informed consent to participate in this study.

## Author Contributions

CC, VM, DG, BB, FM, and VC conceived the project. CC and VM designed the database and eCRF. CC, SK, DD, and DG recruited patients, collected the specimen and the clinical data. NA and VC designed the study, and optimized the experimental protocol. AP and AG were responsible for the initial handling of the blood samples. AP, AG, and NA performed the experiments. NA acquired, analyzed the data, and performed the statistical analysis. NA, J-PV, FM, and VC interpreted the data. VC supervised the work. NA drafted the manuscript. NA, J-PV, FM, and VC substantially revised the manuscript. J-PV, FM, VC, BB, and CC critically reviewed the manuscript. All the authors approved the final manuscript as submitted.

## Funding

This work was supported by a grant “special-COVID-19” from the Université Libre de Bruxelles (U.L.B.), by the Fonds Erasme, and by the Fondation Jaumotte-Demoulin. NA was supported partially by a U.L.B. grant and partially by a grant from the Fond Erasme, and VC was supported by a COVID-19-U.L.B. grant.

## Conflict of Interest

The authors declare that the research was conducted without any commercial or financial relationships that could be construed as a potential conflict of interest.

## Publisher’s Note

All claims expressed in this article are solely those of the authors and do not necessarily represent those of their affiliated organizations, or those of the publisher, the editors and the reviewers. Any product that may be evaluated in this article, or claim that may be made by its manufacturer, is not guaranteed or endorsed by the publisher.

## Abbreviations

AUC, Area under the curve; CD, Cluster of Differentiation; DC, dendritic cells; IL, Interleukin; IL-22BP, interleukin-22 binding protein; IL-22R, interleukin-22 receptor; mDC, myeloid dendritic cells; pDC, plasmacytoid dendritic cells; ROC, Receiver operating characteristics; PBMC, peripheral blood mononuclear cells; WB, whole blood.
