# Supplementary material for: Distinct Expression Patterns of Interleukin-22 Receptor 1 on Blood Hematopoietic Cells in SARS-CoV-2 Infection
Source: Front Immunol. 2022 Mar 29;13:769839. doi: 10.3389/fimmu.2022.769839 (PMC9004465; doi:10.3389/fimmu.2022.769839)
Supplement: Supplementary file 1 [file DataSheet_1.docx]

# **Supplementary Table 1.**

Antibody clones and fluorochromes for phenotypic characterization of innate cells and functional characterization of NK cells and T lymphocytes.

| Specificity | Clone | Fluorochrome | Company | Catalogue number |
| --- | --- | --- | --- | --- |
| Panel A. Phenotypic characterization | | | | |
| CD45 | HI30 | EF450 | Invitrogen | 48-0459-42 |
| CD3 | UCHT1 | EF506 | Invitrogen | 69-0038-42 |
| CD14 | 61D3 | FITC | Invitrogen | 11-0149-42 |
| CD16 | EBIOCB16 | SB780 | Invitrogen | 78-0168-42 |
| CD123 | 6H6 | SB600 | Invitrogen | 63-1239-42 |
| CD11c | 3.9 | SB645 | Invitrogen | 64-0116-42 |
| CD141 | JAA17 | PE-Cy7 | Invitrogen | 25-1419-42 |
| CD56 | CMSSB | APC eFluor780 | Invitrogen | 47-0567-42 |
| HLA-DR | LN3 | SB702 | Invitrogen | 67-9956-41 |
| IL-22R | 305405 | PE | R&D Systems | FAB2770P |
| Panel B. Functional characterization | | | | |
| CD3 | UCHT1 | EF506 | Invitrogen | 69-0038-42 |
| CD4 | OKT-4 | APC-Cy7 | Biolegend | 317418 |
| CD8 | SK1 | BV605 | BD Bioscience | 564116 |
| CD14 | M5E2 | AF700 | Biolegend | 301822 |
| CD16 | EBI0BCB | SB780 | Invitrogen | 78-0168-42 |
| CD56 | CMSSB | APC | Invitrogen | 17-0567-42 |
| IL-22R |  | PE | R&D Systems | FAB2770P |
| IL-22 | URTI | PE-Cy7 | Invitrogen | 25-7229-42 |
| IL-17A | BL168 | FITC | Biolegend | 512303 |
| IFN-γ | B27 | BV711 | BD Bioscience | 564039 |
| GranzymeB | GB11 | BV421 | BD Bioscience | 563389 |
| Perforin | B-D48 | PerCP Cy5.5 | Biolegend | 353314 |

# **Supplementary Table 2.**

Plasma proinflammatory cytokines and chemokines concentrations in healthy controls (n=6), non-severe (n=11) and severe (n=10) COVID-19.

|  | Healthy | *p* values Healthy *vs* non-severe | Non-severe | *p* values Non-severe *vs* severe | Severe | *p* values Healthy *vs* severe |
| --- | --- | --- | --- | --- | --- | --- |
| IL-6 | 1 pg/mL (0.75-2) | **ns** | 8 pg/mL (2-20) | **ns** | 26,5 pg/mL (15-143.8) | ******* |
| IFN-α | 37 pg/mL (21-50) | ****** | 126 pg/mL (103-192) | **ns** | 156,5 pg/mL (104-213.3) | ****** |
| IFN-γ | 2 pg/mL (0.75-3.3) | **ns** | 10 pg/mL (2-13) | **ns** | 9 pg/ml (7-25.8) | ****** |
| IL-1β | 0 pg/mL (0-15) | ***** | 20 pg/mL (18-40) | **ns** | 24 pg/mL (17-47) | ***** |
| TNF-α | 21 pg/mL (16.8-25.8) | ***** | 57 pg/mL (28-74) | **ns** | 48,5 pg/mL (29.8-62.3) | ***** |
| IL-10 | 0 pg/mL (0-0) | **ns** | 10 pg/mL (0-22) | **ns** | 22 pg/mL (11.8-61.8) | ****** |
| IP-10 | 136 pg/mL (97.3-165.5) | **ns** | 1796 pg/mL (649-8922) | **ns** | 8107 pg/mL (1515-34789) | ****** |
| MIG | 1275 pg/mL (1038-2428) | **ns** | 2226 pg/mL (1046-3960) | **ns** | 7446 pg/mL (2945-10022) | ***** |
| MCP-1 | 224 pg/mL (155-257) | **ns** | 280 pg/mL (208-525) | **ns** | 505 pg/mL (429-548.5) | ****** |

Kruskal Wallis test was used to compare results obtained in the different groups. Resuts are indicated as non-significant (ns), or by the degree of significance of the comparison (p). *p<0.05, **p<0.01 and ***p<0.001. IFN-γ, interferon gamma; IL, interleukin; IP-10, interferon gamma-induced protein 10; MCP, monocyte chemoattractant protein; MCP, monocyte chemoattractant protein; MIG, Monokine induced by gamma interferon; TNF-α, tumor necrosis factor alpha.

# **Supplementary Figure 1.**

**
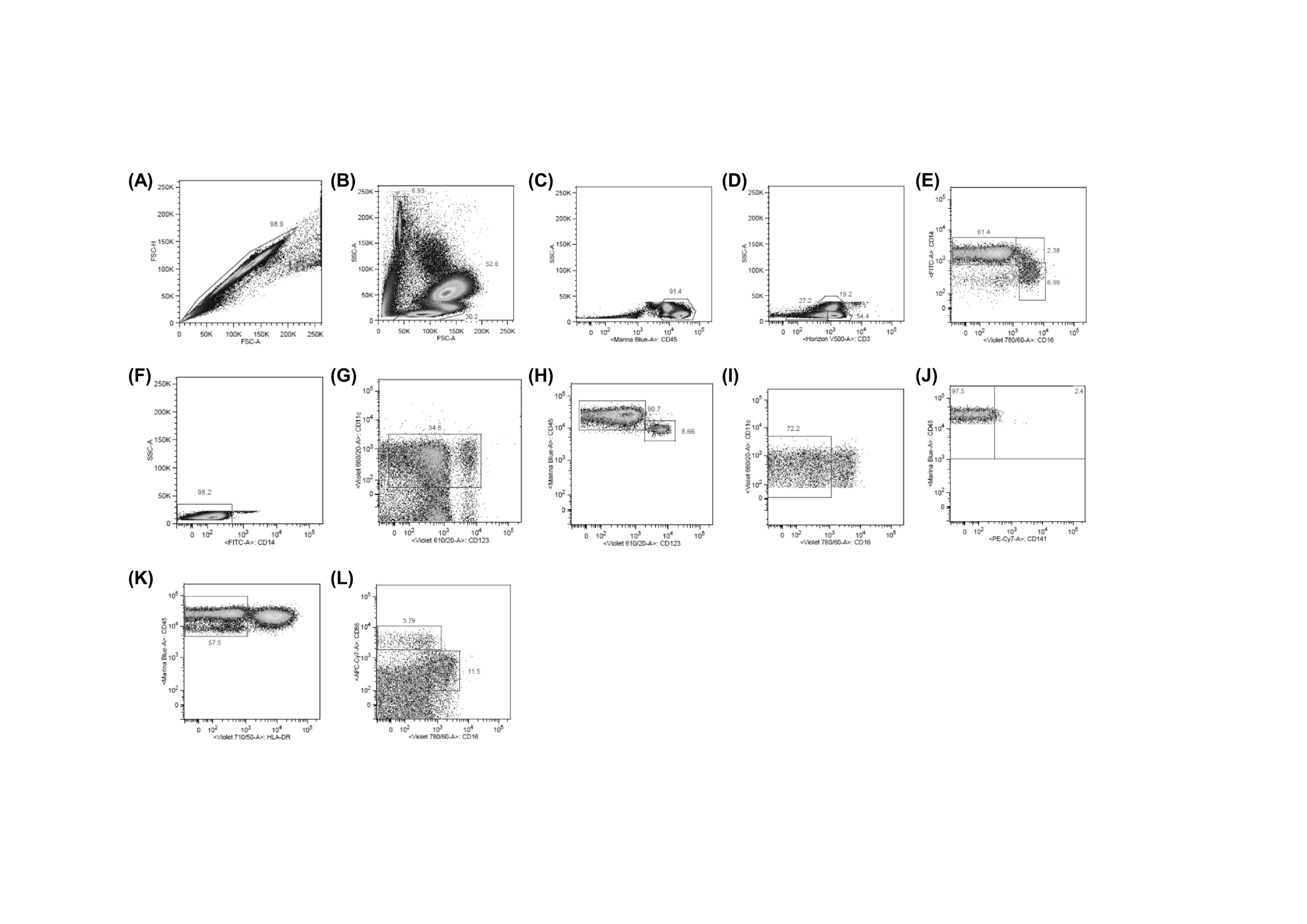
**

**Supplementary Figure 1.** Gating strategy for phenotypic characterization of the monocytes, dendritic cells (DC), NK cells, and their cell subsets obtained from whole blood of a healthy representative volunteer. **(A)** Alive and single cells were selected after doublet exclusions by plotting forward scatter height and forward scatter area. **(B)** Mononuclear cells region was selected based on forward scatter and side scatter area. **(C)** Mononuclear cells were further identified on a CD45 dot plot. **(D)** A side scatter area / CD3 dot plot was used to define the main monocyte population, CD3^+^ lymphocyte and CD3^neg^ cell regions. **(E)** Within the monocytes gate, the monocyte subsets were defined as CD14^high^CD16^neg^ classical monocyte, CD14^high^CD16^pos^ intermediate monocyte and CD14^low^CD16^pos^ non-classical monocyte subsets. Within the CD3^neg^ gate and **(F)** after exclusion of CD14^pos^ cells, **(G)** CD123^pos^ plasmacytoid and CD11c^pos^ myeloid DC subsets were defined on CD123 / CD11c dot plot. **(H)** Plasmacytoid DC were further identified on a CD45 / CD123 dot plot, while **(I)** myeloid cells were further selected on a CD16 / CD11c dot plot. **(J)** Within the myeloid cells gate, CD141^pos^ myeloid DC1 and CD141^neg^ myeloid DC2 were defined on a CD141 / CD45 dot plot. Within the CD3^neg^ cells gate and **(K)** after exclusion of HLA-DR^high^ cells, **(L)** CD56^bright^ and CD56^dim^ NK cell subsets were identified based on a CD16 / CD56 dot plot.

# **Supplementary Figure 2.**

**
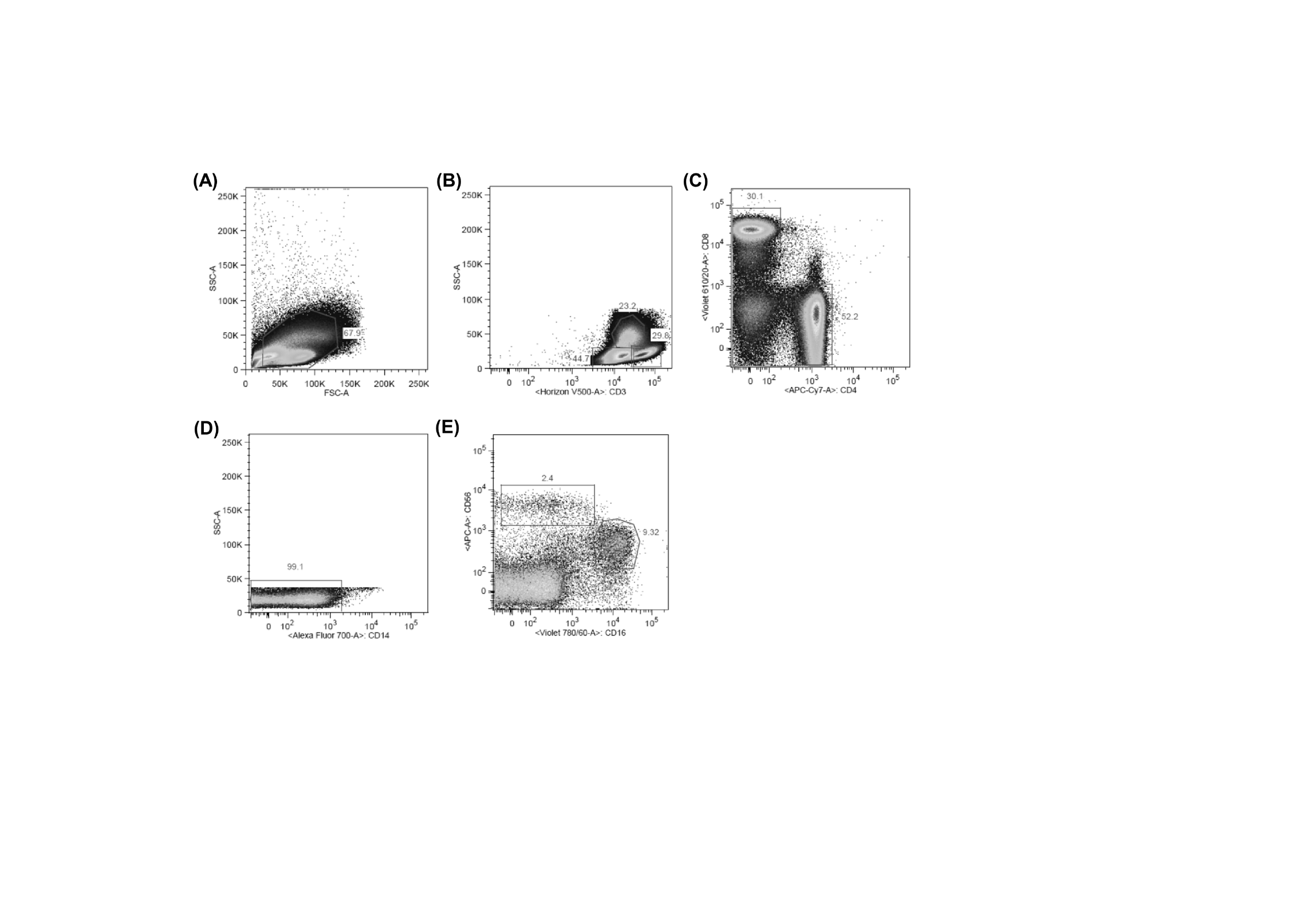
**

**Supplementary Figure 2.** Gating strategy before functional characterization of the NK cells, monocyte subsets and T lymphocytes obtained from PBMC of a healthy representative volunteer. After doublet exclusions by plotting forward scatter height and forward scatter area and selection of alive and single cells, **(A)** PBMC region was determined on forward scatter and side scatter area. **(B)** A side scatter area / CD3 dot plot was used to define monocytes, CD3^+^ lymphocytes and CD3^neg^ cells. **(C)** Within the CD3^+^ lymphocytes gate, CD4^+^ and CD8^+^ T cells were selected on a CD4 / CD8 dot plot. **(D)** Within the CD3^neg^ cells gate, CD14^pos^ cells were first excluded to allow the identification of **(E)** CD56^bright^ and CD56^dim^ NK cell subsets based on a CD16 / CD56 dot plot.

# **Supplementary Figure 3.**


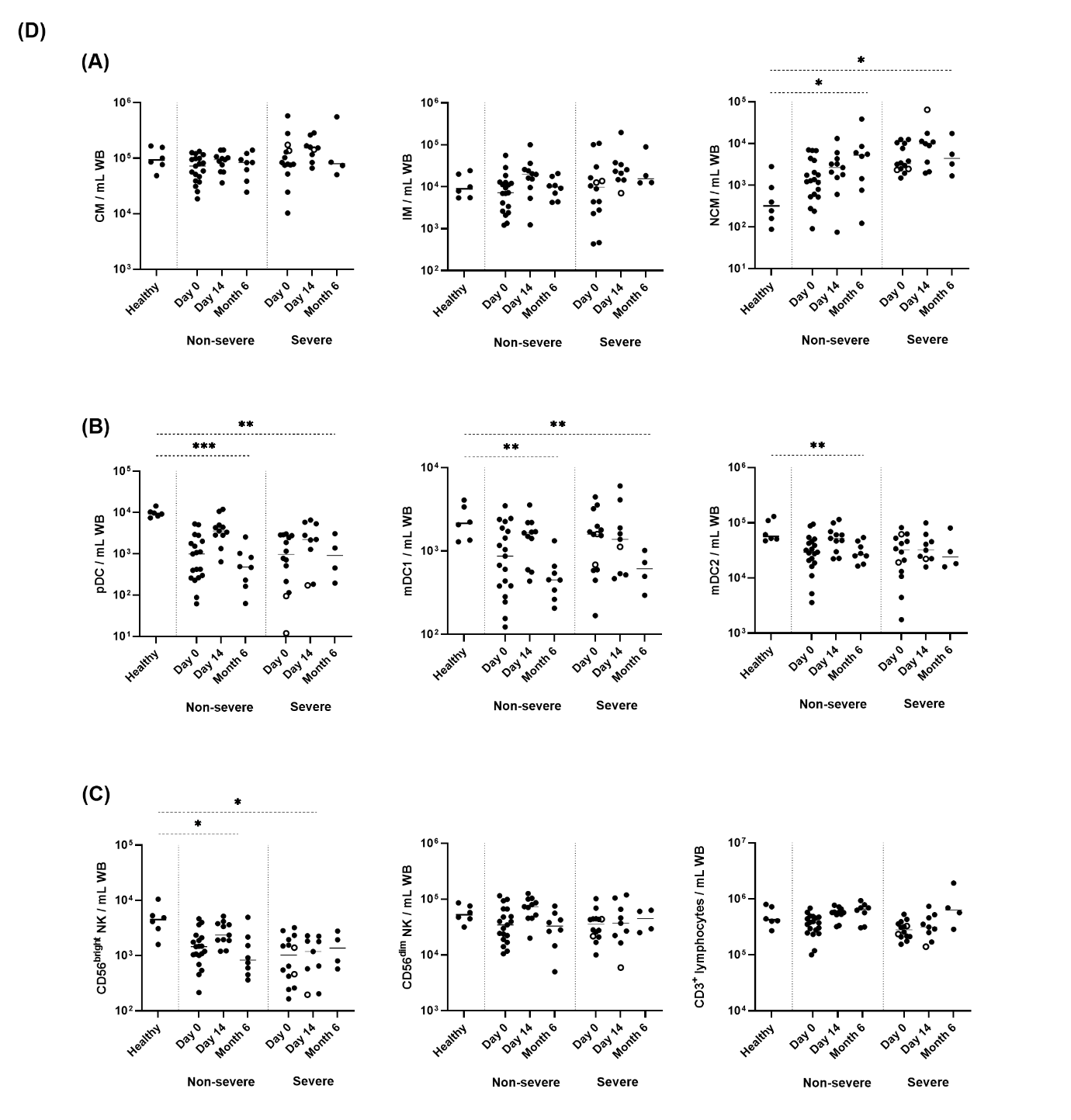


**Supplementary Figure 3.** Kinetic analysis of peripheral blood cells in COVID-19 patients compared to healthy controls. Absolute numbers of **(A)** classical monocytes (CM), intermediate monocytes (IM) and non-classical monocytes (NCM); **(B)** plasmacytoid DC (pDC), myeloid DC1 (mDC1) and myeloid DC2 (mDC2); **(C)** CD56^bright^ and CD56^dim^ NK cells **(D)** CD3^+^ lymphocytes, are represented for healthy controls (n=6), non-severe (n=19) and severe (n=14) COVID-19 patients. Results from patients are shown at day 0, after 14 days (11 non-severe, 9 severe), and six months later (8 non-severe, 4 severe). The patients who died from COVID-19 are represented on the graphs as empty circles. The horizontal bars indicate the medians of the results within each column. Mann Whitney U tests were performed to compare results obtained for patients at 6 months to those from healthy controls and significant differences are represented as **p*<0.05, ***p*<0.01, ****p*<0.001. Time point comparisons for the patients at D0, D14 and Month 6 restricted to paired samples were performed by Friedman test (8 non-severe, 4 severe). For DC numbers, significant differences were noticed for non-severe patients for pDC at Month 6 compared to D14 (*p*<0.05), and for mDC1 at Month 6 compared to D0 (*p*<0.05) and to D14 (*p*<0.05). For NK cell numbers, significant differences were noticed for non-severe patients, both for CD56^bright^ and CD56^dim^ NK cells, at D14 compared to D0 (*p*<0.05) and at Month 6 compared to D14 (*p*<0.05).

# **Supplementary Figure 4.**


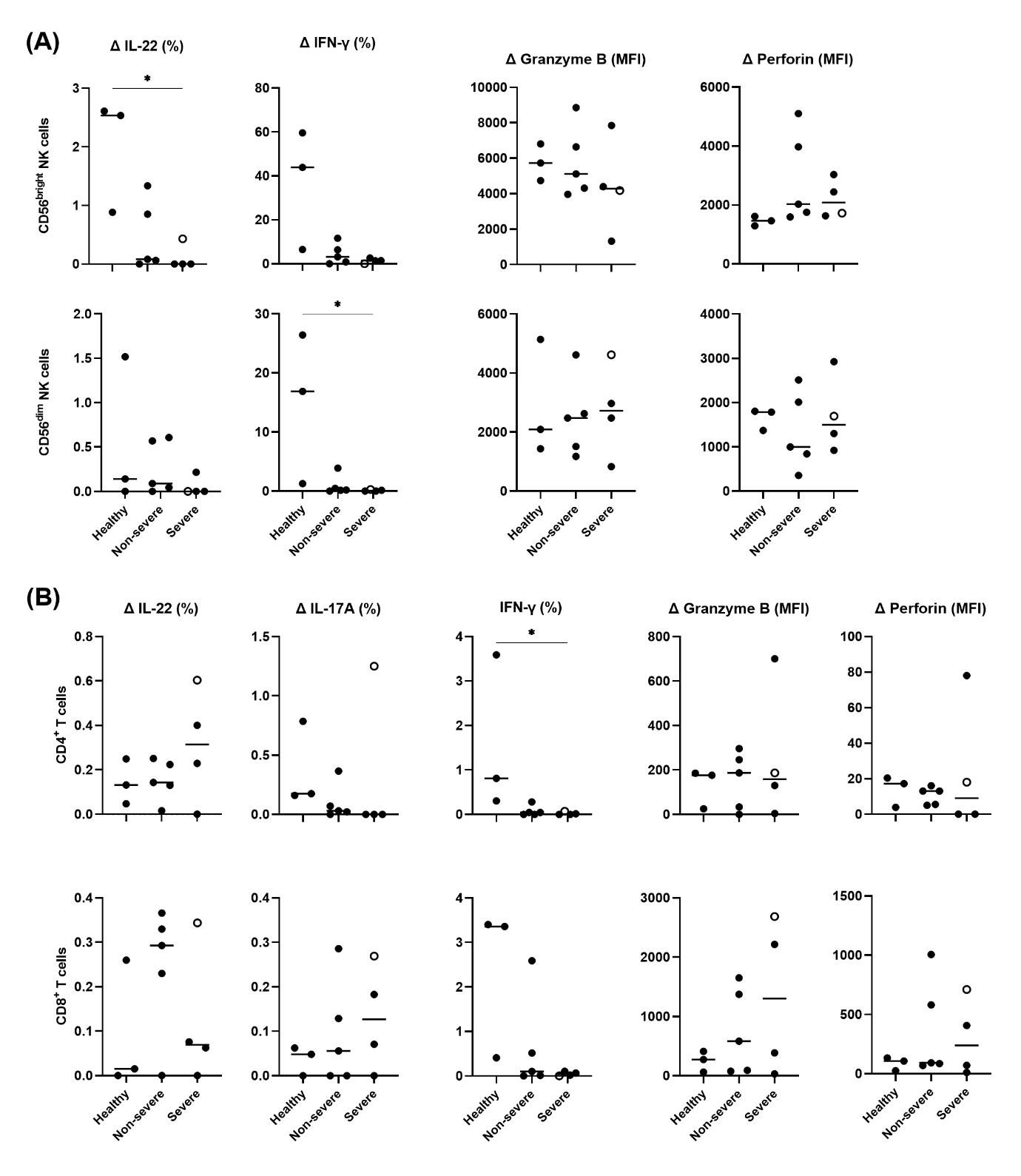


**Supplementary Figure 4.** Cytokines and cytotoxicity mediators contents of peripheral blood NK and T cells after IL-12/15/18 stimulation of PBMC from COVID-19 patients compared to healthy controls. **(A)** IL-22, IFN-γ, granzyme B and perforin expressions are represented for CD56^bright^ and CD56^dim^ NK cells, **(B)** IL-22, IL-17A, IFN-γ, granzyme B and perforin expressions are shown for CD4^+^ and CD8^+^ lymphocytes. The PBMC from 3 healthy controls, 5 non-severe and 4 severe COVID-19 patients were *in vitro* stimulated with IL-12/15/18. The cytokine content of the cells was expressed as percentages of cytokine-containing cells after subtraction of the result obtained for unstimulated cells. The granzyme B and perforin content of the cells was reported as MFI after subtraction of the MFI obtained for unstimulated cells. The patients who died from COVID-19 are represented as empty circles. The horizontal bars indicate the medians of the results within each column. Results obtained for different groups were compared by the Kruskal Wallis test and the significant *p* values < 0.05 (*) are indicated on the graphs.
